# Heat-induced mutator: DNA repair polymerase Polλ shapes mutation under heat

**DOI:** 10.1093/plcell/koag100

**Published:** 2026-04-09

**Authors:** Satoyo Oya

**Affiliations:** Assistant Features Editor, The Plant Cell, American Society of Plant Biologists; Department of Plant Sciences, University of California Davis, Davis, CA 95616, United States; Department of Molecular Biology, Max Planck Institute for Biology Tübingen, Tübingen 72076, Germany

I hope my colleagues are not reading this because I am going to tell you my secret. I accidentally left my DNA samples at 37 °C overnight recently. I had to throw them away because I needed undamaged DNA. What an elementary mistake! We all know that heat is bad for DNA, but look out from your window: DNA is exposed to environmental heat in plants.

Thermal energy induces many DNA lesions, including modified bases, abasic sites, and strand breaks. Unsurprisingly, *Arabidopsis thaliana* accumulates more mutations when propagated at higher temperatures (Belfield et al. 2021; Lu et al. 2021). Heat also boosts CRISPR-based genome editing in euchromatic regions of plants, which has been linked to higher CRISPR cutting activity at elevated temperatures in vitro.

However, a new study by **Clair M. Wootan and colleagues** (Wootan et al. 2026) demonstrates that heat-induced mutagenesis is not a simple consequence of more DNA damage; it strongly reflects how cells handle the damage. The authors showed that, without a single heat-inducible gene (*DNA polymerase λ*, *Polλ*), the mutation rate did not rise under heat. *Polλ* was also responsible for changing the CRISPR-edit outcome upon heat.

First, the authors analyzed how heat boosts CRISPR-Cas9 editing efficiency. They used a CRISPR-Cas9 construct with 2 guide RNAs: one targeting the single-copy gene, and the other targeting an 8-copy sequence distributed across diverse chromatin contexts (Weiss et al. 2022). Without heat, editing efficiency was high at euchromatic sites (30% to 89%) but low at heterochromatic sites (2.0% to 4.1%), as previously known (Fig. 1a). Editing rates rose with increasing heat. With 3 rounds of 37 °C treatment, editing efficiency significantly increased at all targets, including heterochromatin, where rates rose 12.8- to 29.9-fold.

**Figure 1:**
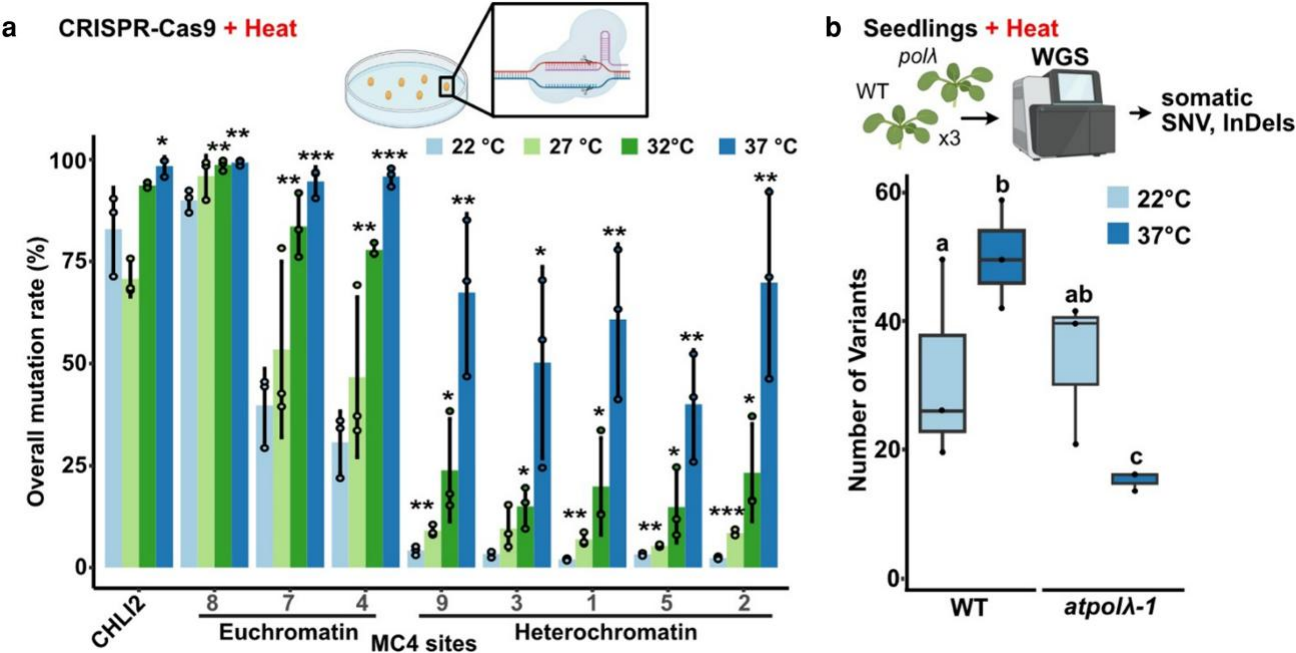
(a) Heat stress increases CRISPR-Cas9 editing rates across diverse chromatin landscapes. (b) DNA *Polλ* mediates genome-wide mutagenesis under heat stress. Comparisons of the total number of de novo SNVs per genotype under heat stress and control conditions. WGS, whole genome sequence; InDels, insertions and deletions. Adapted from Wootan et al. (2026), Figures 1 and 4.

Intriguingly, heat not only increased editing efficiency but also shifted the “type of edits” to 1-bp insertions. Across all target sites, the fraction of 1-bp insertion edits rose 1.2- to 3.6-fold under heat stress. 1-bp insertion at the CRISPR site is a telltale signature of *Polλ* (Weiss et al. 2024), an error-prone polymerase that fills short DNA gaps in multiple DNA-repair pathways, including CRISPR-site repair. Indeed, the *polλ* mutant produced nearly undetectable levels of 1-bp insertions at a cut site, regardless of temperature. In wild type, by contrast, 1-bp insertions were approximately 20% without heat and surged to >50% with heat, indicating that *Polλ* is responsible for the heat-induced shift in editing outcomes. Moreover, *Polλ* expression increased 4.6-fold after heat stress. It was previously known that overexpression of *Polλ* was sufficient to tilt CRISPR outcomes toward 1-bp insertions (Weiss et al. 2024). Together, these results indicate that heat induces *Polλ* and thereby shifts the outcome of double-strand break repair toward a 1-bp insertion.

The authors next asked whether *Polλ* also underlies the genome-wide increase in mutations previously observed under heat. They identified de novo somatic mutations through high-depth whole-genome sequencing of seedlings. As expected, wild type had more single nucleotide variants (SNVs) after heat stress (Fig. 1b). The *polλ* mutant had a similar number of mutations to the wild type under normal conditions, but under heat, it had significantly fewer SNVs and indels. This suggests *Polλ* as a mutator responsible for genome-wide heat-induced mutagenesis.

Why would plants induce *Polλ* expression, risking the genome integrity? One explanation could be that *Polλ* is confined to tissues that do not contribute to the next generation. But reanalysis of the public expression data (under non–heat-stress conditions) suggest otherwise, showing *Polλ*'s prominent expression in SAM. Another explanation could be that *Polλ* is an emergency repair that confer viability under stress at the expense of accuracy. However, under Wootan and colleagues’ condition, *polλ* mutants were not phenotypically heat sensitive, which does not support (though nor conclusively exclude) this idea.

Overall, these results suggest that plants can be programmed to elevate mutation under heat. The authors offer a tantalizing speculation: the induced mutation promotes genetic diversity and the adaptive potential in the offspring. Although whether *Polλ*'s heat inducibility could have evolved for adaptive mutagenesis would be another, hard-to-prove hypothesis, this work raises exciting questions as well as offering practical guidance for genome editing. Could the *Polλ*-driven, heat-induced mutagenesis leave detectable signatures at an ecological scale? Does the reduction of genome-wide mutation rates in the absence of Polλ indicate heat-induced increase of fidelity, which is normally masked by *Polλ*? Finally, the CRISPR assay shows that the *Polλ* dependence somewhat varies with chromatin context, raising the question of whether chromatin will bias *Polλ*-mediated mutagenesis.

Let us speculate. But before putting our heads above the clouds, let us put our DNA sample in the freezer and our CRISPR plants under heat.

## Recent related articles in *The Plant Cell*:

- Quiroz et al. (2024) demonstrated DNA-repair protein MSH6 binds to chromatin mark H3K4me1 and biases mutation in chromatin dependent manner. They also described the somatic mutation-calling method used here.
- Samach et al. (2023) developed a visual assay to identify large-scale consequences of CRISPR-Cas9 outcomes such as loss of chromosomal segments or crossover, which may be tricky to detect by targeted sequences of the cut site.
- Cheng et al. (2025) delivered a suite of 61 plant CRISPR T-DNA vectors, spanning Cas9/SpRY, Cas12a, base editors, and CRISPR activation, designed for 1-step guide or library cloning.

## Data Availability

None to declare.

## Dive Curated Terms

The following phenotypic, genotypic, and functional terms are of significance to the work described in this paper:

MSH6 Gramene: AT4G02070
MSH6 Araport: AT4G02070

Pol? Gramene: AT1G10520
Pol? Araport: AT1G10520
